# JBMR Plus Reviewer list 2023

**DOI:** 10.1002/jbm4.10844

**Published:** 2023-12-19

The ASBMR and the journal Editors are incredibly thankful for the reviewers who volunteer their time for the vital mentoring role of peer reviewer. The following individuals have reviewed for JBMR Plus in the past year and have provided invaluable feedback to help improve submitted papers.

Data through November 14, 2022 – October 25, 2023.

*Indicates 3 or more reviews completed.

**Indicates 5 or more reviews completed.

Annette Adams

Riad Akhundov

Henry Akinbi

Ahmed Al Saedi*

Imranul Alam

Matt Allen

Catherine Ambrose

Natasha Appelman‐Dijkstra

John Asplin*

Reggie Aurora

Ugur Ayturk

Yangjin Bae

Chelsea Bahney

Jeannie Bailey

Rafal Bartoszewski

Kristen Beavers

Sanjay Bhadada

Harry Blair

Robert Blank**

Elizabeth Bradley

Mikkel Brent

Jacques Brown*

Lucas Brun

Bjoern Buehring

Ellish Burke

David B. Burr*

Bjoern Busse

Jay J. Cao

Dana Carpenter

Alesandra Carriero

James Cassat

Naibedya Chattopadhyay

Chien‐Liang Chen*

Jin‐RanChen

Je‐Yong Choi

Cristiana Cipriani

Brian C. Clark

Martine Cohen‐Solal

Adi Cohen**

Mary Cole

Kelsey Collins

Fabio Vasconcellos Comim

Tim Cootes

Sabrina Corbetta

Nicola Crabtree

April Craft

Carolyn Crandall

James Cray*

Laura Creemers

Souad Daamouch

Rafael de Molon

Susan Diem

Naomi Dirckx

Sinee Disthabanchong

Paola Divieti Pajevic*

Kate Duchowny

Emma Duncan

Florent Elefteriou

Klaus Engelke

Daniel Evans

Peter Fabian

Alberto Falchetti*

Charles Farber

Virginia Ferguson

Aníbal Ferreira

Trine Elisabeth Finnes

Antonella Forlino*

Andrea Fox

Morten Frost

Seiji Fukumoto**

Dana Gaddy

Gustavo Garlet

Damian Genetos**

Samuel Ghatan

Charles Ginsberg

Neil Gittoes

David Goldhamer

Corinna Grasemann

Matthew Greenblatt*

Jennifer Gregory

Anne Grethe Jurik

Yaodong Gu*

Scott A. Guelcher

Anyonya Guntur

Melanie Haffner‐Luntzer

Dieter Haffner

Mark Hamrick

Sarah Hardcastle**

Nan Hatch

Alberto Hidalgo‐Bravo

Kevin Hoffseth

Hironori Hojo

Nilsson Holguin

Michael Holick

Kara Holloway‐Kew

Kuo‐Chin Huang

Julie Hughes

Steven Ing*

Alex Ireland

Carlos Isales**

M. Kassim Javaid

Karl Jepsen

Eijiro Jimi

Fjola Johannesdottir*

Mark Johnson

HiroshiKaji

Lamya Karim

Kurt Kennel

Ha‐Neui Kim*

Keunyoung Kim

Frank Ko**

Joyce S.B. Koh

Yoshihiro Komatsu

Sung Hye Kong

Susan Krum

Takuo Kubota

Lisa Langsetmo

Christine Lary

Michael Laurent

Carole Le Henaff*

Maarit Leinonen

William Leslie

Michael Levine

Huiqi Li

Michael T.C. Liang

Cesar Libanati

Eva Liu

Huan Liu

Julian Lui

Guillaume Mabilleau

Heather Macdonald

Jay Magaziner

Outi Makitie*

Kim Mansky

Claudio Marcocci

Takamitsu Maruyama

Yuki Matsumura

Takehiko Matsushita

Yuki Matsushita

Gherardo Mazziotti

Gabriel Mbalaviele

Michael McClung

Meghan McGee‐Lawrence**

Donal McNally

Robert Meertens

Daniela Merlotti*

Lisa K. Micklesfield

Salvatore Minisola*

Takeshi Miyamoto

SharonMoe

Pierre Moffatt

Subburaman Mohan

Rebecca Moon

Roy Morello

Kendall Moseley*

Katherine Motyl

Christian Muschitz

Sandesh Nagamani

Mark Nanes

Thomas Nickolas

Keizo Nishikawa

RobertNissenson

Jeffry S. Nyman

Mícheál Ó Breasail

Ralf Oheim

Catherine Omosule

Noriaki Ono*

Wanida Ono

Benjamin Osipov

Miguel Otero

Neil Paloian

Serk In Park

Eleftherios Paschalis*

Erwin Pauws

John Pettifor

Marcelo Pinheiro

Lilian Plotkin**

IgnacioPortales Castillo

Sudhaker Rao

Alexander Rauch

Frank Rauch

Martina Rauner

Revit Regev

Ian Reid

R. Revuelta‐Iniesta

Yumie Rhee

Amy Ribet

Alexander Rodriguez

Tim Rolvien

Genevieve Romanowicz

Tamara Rozental

Mishaela Rubin

Eric Rush

Danielle Rux

Nithin S

Khashayar Sakhaee

Amy Sato

Dominik Saul

Mitchell Schaffler

Erica Scheller

Inez Schoenmakers

Ann Schwartz*

Peter Schwarz

David Scott

Ego Seeman

Nicole Sekel

Deborah E. Sellmeyer**

Jad Sfeir**

John Shepherd

Matthew Silva

Ravinder Singh

Analuiza Soares

Anne Sophie Sølling

Lauren Surface*

Rachel Surowiec

Taiki Suzuki

Pawel Szulc**

Hanna Taipaleenmäki

Masahiko Takahata

Yoshifumi Takahata

Michaela Tencerova

Jesper Thomsen

Dwight Towler

Philip Trackman

Elena Tsourdi**

Masayuki Tsukasaki

Nobuyuki Udagawa

Yasuyoshi Ueki

Piyush Uniyal

Wim Van Hul

Rafael Velazquez‐Cruz

Anja Verhulst

Charlotte Verroken

Peter Vestergaard**

Tatiane Vilaca*

Yadav Wagley

W. Angus Wallace

Matthias Walle

Bowen Wang

Jialiang Wang

Leanne Ward

Ashley Weaver**

Connie Weaver

David Weber

Thomas Weber

Marc Wein

Deborah Wenkert

Robert Wermers

Jennifer Westendorf

Sarah Wherry*

Thomas L. Willett

BettinaWillie

Karen Winer

Eva Wölfel*

Danielle Wu*

Feitong Wu*

Joy Wu

Jinhu Xiong

Chao Xu

Tao Yang

Pamela Yelick

Clare Yellowley

Timur Yorgan

Sebastian Zanner

Babette Zemel

Cristiano Zerbini

Hongyuan Zhang

Bo O. Zhou

Heinz Zoller*

